# Gallstones were associated with the gastrointestinal adverse events of cinacalcet in hemodialysis patients with secondary hyperparathyroidism

**DOI:** 10.1080/0886022X.2017.1419971

**Published:** 2018-01-04

**Authors:** Keiichi Otsuka, Yoichi Ohno, Joji Oshima

**Affiliations:** a Department of Internal Medicine, Oshima Clinic, Saitama, Japan;; b Department of Nephrology, Saitama Medical School, Saitama, Japan

**Keywords:** Cinacalcet hydrochloride, gallstones, gastrointestinal adverse events, hemodialysis, secondary hyperparathyroidism

## Abstract

This study aimed to investigate the association of gastrointestinal (GI) adverse events of cinacalcet with gallstones in the hemodialysis (HD) patients with secondary hyperparathyroidism (SHPT). A total of 23 HD patients under the treatment with cinacalcet and 101 control patients were enrolled in this cross-sectional study. We investigated the prevalence of gallstones and the association of GI adverse events of cinacalcet with gallstones. The prevalence of gallstones was significantly higher in the HD patients with cinacalcet compared with the controls (47.8% vs. 15.8%). The longer time on HD, hypercalcemia, hyperphosphatemia and elevated parathyroid hormone level were observed in the HD patients with cinacalcet. Besides, GI adverse events of cinacalcet were observed more frequently in the HD patients with gallstones compared with those without gallstones (odds ratio 13.5, 95% CI: 1.80–101). Therefore, screening for gallstones before dosing cinacalcet may reduce the risk of GI adverse events in SHPT patients.

## Introduction

Chronic kidney disease-mineral and bone disorders (CKD-MBD) have been associated with poor health outcomes, including diminished quality and length of life. Vitamin D deficiency and secondary hyperparathyroidism (SHPT) play a major role on progressing CKD-MBD stage. Standard management for CKD-MBD includes phosphate restricted diet, vitamin D and phosphate binders. SHPT refers to the excessive secretion of parathyroid hormone (PTH) by the parathyroid glands in response to hypocalcemia and associated hyperplasia of the glands. SHPT is a common complication that develops early in CKD and progresses rapidly in end-stage renal disease [1]. Persistently elevated PTH levels may require the addition of calcimimetics such as cinacalcet hydrochloride (cinacalcet) especially in the patients on long-term maintenance hemodialysis (HD).

Cinacalcet activates the parathyroid cell calcium-sensing receptor (CaSR) and inhibits PTH secretion [2] and simultaneously lowers serum phosphorus and calcium level in the HD patients with SHPT [3]. The most commonly reported adverse events were digestive symptoms such as nausea and vomiting [3]. However, the underlying mechanism of the gastrointestinal (GI) adverse events induced by cinacalcet remains to be elucidated. Recently, we happened to encounter two consecutive cases of acute cholecystitis in the HD patients under the treatment for SHPT with cinacalcet. Firstly, we aimed to investigate the prevalence of gallstones in the HD patients under the treatment with cinacalcet. Secondly, we investigated the relationship between GI adverse events of cinacalcet and gallstones in the HD patients with SHPT.

## Patients and methods

A total of 23 HD patients under the treatment with cinacalcet (cinacalcet group) and 101 HD patients without cinacalcet (control group) in our HD unit were enrolled in this cross-sectional study. Written informed consents were taken from all the study participants. Detailed medical history was taken in all patients, and the usage and the GI adverse events of cinacalcet were confirmed in the cinacalcet group by inquiry and charts. Cinacalcet was prescribed only in the HD patients under poor control of SHPT with vitamin D and phosphate binders. GI adverse events were defined as nausea that occurred repeatedly after the initial dose (25 mg per day) or the increased dose of cinacalcet in the present study. Most of the patients tolerated nausea by cinacalcet as time passed except the patients complicated with acute cholecystitis. Physical examination and abdominal ultrasonography were conducted to confirm stones and polyps of gallbladder. Baseline continuous variables such as age, time on HD and laboratory values were compared between the cinacalcet group and the control group, and also compared between the patients of the cinacalcet group with or without GI adverse events. The relations of categorical variables such as sex, regularly prescribed antacids, history of upper GI diseases, gallbladder polyps and gallstones to the usage of cinacalcet were evaluated by calculating odds ratio (OR) in all HD patients. The relations of these categorical variables to the incidence of GI adverse events were also estimated by calculating OR in the cinacalcet group. The ethics committee of the institute approved this study. An ‘opt-out’ method was adopted and explained to the patients where they were informed of their inclusion in the study.

### Statistical analysis

All statistical analyses were performed by SPSS software (Chicago, IL). Continuous variables were reported as mean ± standard deviation (SD) and compared using Student’s *t*-test. Categorical variables were reported as percentages and compared by calculating OR. A *p* value of less than .05 was considered to be statistically significant.

## Results

As shown in Table 1, the mean age of the cinacalcet group was younger than that of the control group (62 vs. 68 years, *p* < .05). However, the time on HD was significantly longer in the cinacalcet group compared with the control group (13.6 vs. 5.4 years, *p* < .001). Dry weight, serum alkaline phosphatase (ALP), hemoglobin, ferritin, total protein and blood urea nitrogen (BUN) were similar in both the groups. Whereas, corrected Ca, phosphorus, whole PTH, creatinine, Kt/V, albumin, potassium, low-density lipoprotein cholesterol (LDL-C) and β2-microglobulin (MG) were significantly higher in the cinacalcet group compared with the control group. Transferin saturation (TSAT) was lower in the cinacalcet group than the control group. As shown in Table 2, there was no association of sex, regularly prescribed antacids, history of upper GI diseases and gallbladder polyps with the usage of cinacalcet. However, the prevalence of gallstones was 47.8% in the cinacalcet group, significantly higher than that of the control group: 15.8% (OR = 4.87, 95% CI: 1.84–12.9).

**Table 1.** Baseline continuous variables of the study groups.

	Cinacalcet group, *N* = 23	Control group, *N* = 101	*p* value	Cinacalcet group with AE, *N* = 12	Cinacalcet group without AE, *N* = 11	*p* value
Age (years)	62 ± 10	68 ± 11	.017	64 ± 8	60 ± 10	.291
Time on HD (years)	13.6 ± 4.9	5.4 ± 5.2	<.001	13.6 ± 4.4	13.5 ± 5.1	.986
Dry weight (kg)	57.3 ± 9.8	54.8 ± 12.9	.380	53.7 ± 7.0	61.2 ± 10.4	.060
Corrected Ca (mg/dL)^a^	9.3 ± 0.7	9.0 ± 0.7	.036	9.2 ± 0.6	9.3 ± 0.7	.673
Phosphorus (mg/dL)	5.6 ± 1.4	5.0 ± 1.2	.048	5.9 ± 1.5	5.2 ± 1.0	.230
ALP (IU/L)	228 ± 86	265 ± 127	.187	275 ± 91	176 ± 22	.003
Whole PTH (pg/mL)	120 ± 72	87 ± 51	.047	113 ± 23	127 ± 61	.646
Hemoglobin (g/dL)	11.3 ± 1.0	11.1 ± 0.9	.341	11.5 ± 0.7	11.1 ± 1.2	.419
TSAT (%)	19.8 ± 8.8	25.9 ± 11.4	.019	19.7 ± 7.4	20.0 ± 9.8	.925
Ferritin (ng/mL)	101 ± 162	162 ± 268	.293	142 ± 204	55 ± 54	.203
Total protein (g/dL)	6.5 ± 0.4	6.4 ± 0.5	.864	6.5 ± 0.5	6.4 ± 0.4	.379
Albumin (g/dL)	3.9 ± 0.2	3.8 ± 0.3	.011	4.0 ± 0.3	3.9 ± 0.2	.964
BUN (mg/dL)	60.1 ± 11.6	55.2 ± 13.9	.122	60.2 ± 13.0	59.9 ± 9.2	.937
Creatinine (mg/dL)	12.0 ± 2.8	9.5 ± 2.4	<.001	11.3 ± 2.6	12.9 ± 2.7	.177
Kt/V^b^	1.6 ± 0.3	1.5 ± 0.3	.039	1.67 ± 0.16	1.59 ± 0.35	.541
Potassium (mEq/L)	5.2 ± 0.7	4.8 ± 0.8	.044	5.2 ± 0.7	5.1 ± 0.7	.664
LDL-C (mg/dL)	96 ± 26	81 ± 26	.013	98.1 ± 21.0	93.7 ± 29.6	.700
β2-MG (mg/L)	27.9 ± 3.7	25.9 ± 6.2	.044	28.2 ± 4.0	27.6 ± 3.2	.745

Values are mean ± SD; AE: adverse events.

a Corrected Ca = serum calcium + (4-serum albumin); ALP: alkaline phosphatase; PTH: parathyroid hormone; TSAT: transferrin saturation; BUN: blood urea nitrogen; LDL-C: low-density lipoprotein cholesterol; β2-MG: β2-microglobulin.

b Kt/V calculation is Daugirdas’s formula (1996): spKt/V = *−*ln(*R* *−* 0.008 × *t*) + (4 − 3.5 × *R*) 0.55 × *UF*/*V*: in which *R* is predialysis urea/postdialysis urea, *t* is dialysis time in hours.

**Table 2.** The relations of the categorical variables to the usage of cinacalcet.

Categorical variables	Cinacalcet group, *N* = 23	Control group, *N* = 101	Odds ratio	95% CI
Sex (male)	65.2% (15/23)	63.4% (64/101)	1.08	0.42–2.80
Antacids	82.6% (19/23)	66.3% (67/101)	2.41	0.76–7.65
History of upper GI diseases	39.1% (9/23)	23.8% (24/101)	2.06	0.79–5.36
Gallbladder polyps	34.8% (8/23)	35.6% (36/101)	0.96	0.37–2.49
Gallstones	47.8% (11/23)	15.8% (16/101)	4.87	1.84–12.9

In the cinacalcet group, there was no significant difference in the dosages of cinacalcet (40.6 vs. 30.7 mg per day, *p* = .26), Ca-containing phosphate binders (precipitated calcium carbonate 2292 vs. 3455 mg per day, *p* = .20) and intravenous vitamin D (calcitriol 2.0 vs. 1.7 µg per week, *p* = .24) between the patients with or without adverse events. Meanwhile, only ALP was significantly higher in the patients with adverse events than those without adverse events in the cinacalcet group (Table 1). As shown in Table 3, there was no association of sex, regularly prescribed antacids, history of upper GI diseases and gallbladder polyps with the incidence of GI adverse events in the cinacalcet group. However, the GI adverse events of cinacalcet were observed more frequently in the HD patients with gallstones compared with those without gallstones. The OR at which GI adverse events appeared was 13.5 (95% CI: 1.80–101) in the HD patients with gallstones. The details of regularly prescribed antacids and upper GI diseases in the cinacalcet group were as follows. Sixteen patients were on the treatment with proton pump inhibitor, three patients with H2 blocker and four patients without antacids. Six cases of gastric ulcers, a case of duodenal ulcer, three cases of reflux esophagitis and two cases of gastritis were confirmed. No GI prokinetic agent was prescribed regularly in the cinacalcet group.

**Table 3.** The relations of the categorical variables to the incidence of GI adverse events of cinacalcet.

Categorical variables	Cinacalcet group with adverse events, *N* = 12	Cinacalcet group without adverse events, *N* = 11	Odds ratio	95% CI
Sex (male)	66.7% (8/12)	63.6% (7/11)	1.14	0.21–6.37
Antacids	75% (9/12)	90.9% (10/11)	0.30	0.03–3.43
History of upper GI diseases	50.0% (6/12)	27.3% (3/11)	2.67	0.47–15.3
Gallbladder polyps	41.7% (5/12)	27.3% (3/11)	1.91	0.33–11.0
Gallstones	75% (9/12)	18% (2/11)	13.5	1.80–101

## Discussion

Gallstones are crystalline structures formed by concentration or accretion of normal or abnormal bile constituents. Cholelithiasis is a common GI problem, but rarely causes HD patients to admit to hospital compared with heart disease, pneumonia and cerebrovascular disease. The prevalence of cholelithiasis is approximately 10–15% in developed countries [4] and tends to be increased with the advancement of CKD stage [5]. Although the risk of gallstones formation disappeared in HD patients, the prevalence of cholelithiasis was still significantly higher than in the control population [5]. Kazama et al. [5] also summarized the previous reports regarding the prevalence of gallstones in end-stage CKD patients. Several papers reported that the incidence of gallstones in HD patients was similar to that in the controls. Whereas, others have reported high prevalence of cholelithiasis in HD patients from 18.2% to 34.6% [5–8]. To our knowledge, Gençtoy et al. [8] reported the highest prevalence of cholelithiasis as 34.6% that was positively correlated with diabetes, age, number of blood transfusions, high serum phosphorus, ALP and LDL-C level. In the present study, the prevalence of gallstones was 21.8% (27/124) in HD patients similar to 22.9% in the Japanese study previously reported [5]. Since the gallbladder is innervated by the autonomic nervous system, autonomic neuropathy due to uremia may cause gallbladder stasis and develop cholelithiasis in HD patients [9,10]. Our baseline laboratory values such as serum corrected Ca, phosphorus, ALP, LDL-C and whole PTH levels were not significantly different between the HD patients with gallstones and those without gallstones (data not shown). Whereas, the time on HD was significantly longer in the HD patients with gallstones compared with those without gallstones (9.3 vs. 6.2 year, *p* < .05). In the present study, the prevalence of gallstones was high (47.8%) in the HD patients complicated with SHPT treated with cinacalcet (Table 2). The longer time on HD, the higher serum creatinine, LDL-C and β2-MG levels might be associated with the high prevalence of gallstones in the cinacalcet group compared with the control group (Table 1). As far as we know, there was no report that examined the prevalence of cholelithiasis in SHPT patients on HD. We found only one article that showed higher prevalence of cholelithiasis in peritoneal dialysis patients with SHPT [11]. In their study, cholelithiasis was detected in 25% of patients with high PTH level compared with 2.6% of patients with normal PTH level. Female gender, low creatinine, high phosphorus and high PTH levels might be factors increasing the formation of gallstones [11]. In general, SHPT patients show significantly elevated PTH and phosphorus levels in serum. In addition, serum calcium levels tend to be high normal, since both vitamin D and Ca-containing phosphate binders have been usually prescribed or actively prescribed in SHPT patients to avoid hypocalcemia induced by cinacalcet. Similarly, our baseline laboratory values such as serum corrected Ca, phosphorus and whole PTH levels were significantly higher in the cinacalcet group compared with the control group (Table 1). Consequently, the longer time on HD and increased calcium phosphate product facilitates the development of ectopic calcification that may increase the incidence of gallstones in SHPT patients on HD.

On the other hand, primary hyperparathyroidism (PHPT) is usually caused by a tumor within the parathyroid gland. The symptoms of the condition relate to the high serum calcium and PTH levels. PHPT has been considered a rare illness characterized by bone disease, urinary calculi, hypertension, mental disturbances, myopathy, peptic ulcer disease, pancreatitis and cholelithiasis [12]. Cholelithiasis has been reported as a common complication of PHPT [13,14]. Broulik et al. [13] reported that 30.3% of the female and only 8.66% of the male PHPT had gallstones. The gender difference might be because PHPT is considerably more common in female. In general, gallstone prevalence increases with age and gallstones are more prevalent in females of any age group as reported [15]. In the present study, 24.4% (11/45) of female HD patients and 20.3% (16/79) of male HD patients had gallstones. Hypercalcemia is known to decrease bile flow and increase biliary ionized calcium concentration in cats and prairie dogs [16,17]. Similar effects of hypercalcemia on bile composition in humans might promote calcium salt precipitation in bile that may lead to the formation of gallstones. Furthermore, PTH is known as an inhibitor of smooth muscle contraction in the cardiovascular system and the GI tract [18]. The mechanism of PTH-associated gallstone formation may involve inhibition of gallbladder emptying, hepatic bile secretion and Oddi’s sphincter motility as well as modification of bile composition. Although the pathogenesis of gallstones in PHPT remains unclear, several factors including hypercalcemia, elevated PTH level, impaired contractility of the gallbladder and changes in bile composition may be implicated as the cause of cholelithiasis in PHPT. Accordingly, female gender, hypercalcemia and elevated PTH level are common factors in the pathogenesis of gallstones in PHPT and SHPT treated with vitamin D and Ca-containing phosphate binders.

Cinacalcet has been recently considered to cause digestive symptoms by decreasing GI motility *via* activating the CaSR in gut, since human antral gastrin cells express the CaSR [19]. The activation of the CaSR increases serum gastrin level and basal gastric acid secretion in healthy adults [20], whereas cinacalcet delayed GI motility associated with elevated serum gastrin concentration that was independent of gastric acid secretion in HD patients [21]. On the other hand, there was no association of antacids and past history of upper GI diseases with the GI adverse events of cinacalcet in the present study (Table 3). The CaSR has been also implicated in mediating cholecystokinin (CCK) secretion [22]; however, an acute dose of cinacalcet exerts minimal influence on GI hormonal responses to a mixed meal in dialysis patients on chronic therapy with this drug [23]. Intraduodenal calcium is also reported to stimulate pancreatic enzyme secretion and gallbladder contraction in a dose-related fashion, achieving comparable responses to those produced by intravenous CCK [24]. Therefore, both oral intake of cinacalcet and Ca-containing phosphate binders could evoke contraction of gallbladder *via* CCK secretion. In the present study, the GI adverse events of cinacalcet were observed more frequently in the HD patients with gallstones compared with those without gallstones (Table 3). The longer time on HD might be associated with the high prevalence of gallstones in the cinacalcet group, however the time on HD were similar between the patients of the cinacalcet group with or without GI adverse events (Table 1). Besides, there was no significant difference of the time on HD between the patients of the cinacalcet group with or without gallstones (14.6 vs. 12.6 year, *p* = .32). These results suggest that the GI adverse events were independent of the time on HD. In addition, ALP level was significantly higher in the patients of the cinacalcet group with GI adverse events than those without GI adverse events, which may reflect the biliary disorder (Table 1). In general, cinacalcet simultaneously lowers serum PTH, phosphorus and calcium levels in SHPT patients on HD. Since hypocalcemia is known to induce Oddi’s sphincter spasm of dogs [25], cinacalcet may induce gallbladder contraction *via* GI hormones and simultaneously trigger spasm of Oddi’s sphincter *via* hypocalcemia, which can develop the GI adverse events in the SHPT patients with gallstones. In other words, cinacalcet may induce GI adverse events by biliary dyskinesia that is a dynamically obstructive, pain-producing disorder.

## Conclusions

The pathogenesis of gallstones in SHPT patients on HD is unclear. However, we suggest that the longer time on HD, hypercalcemia, hyperphosphatemia and elevated PTH level may be associated with the high prevalence of gallstones in the HD patients with advanced SHPT on cinacalcet. Besides, cinacalcet may frequently induce GI adverse events by biliary dyskinesia in the SHPT patients with gallstones. Therefore, we propose that SHPT patients should be screened for gallstones by ultrasonography before dosing cinacalcet to reduce the risk of GI adverse events.

### Limitations of our study

The present study was primarily designed as a case-control study to determine the association of the GI adverse events of cinacalcet with gallstones. The study participants were not enough and restricted to the one HD unit in a rural Japanese region. Therefore, a large randomized prospective clinical trial will be required to lead to a final conclusion.
